# 

**Published:** 1892-04

**Authors:** 


CONTENTS—APRIL, 1892.—VOL. VII, No. 10.

ORIGINAL ARTICLES—

The Management of Abortions. By J. W. Carhart, M. D. ... 251

Double Amputation of Lower Extremities for Gangrene following Frost Bite. H. B. Hill, M. D., Austin ... 355

Texas Fever and Ticks. B. A. Rogers, M. D ... 357

SOCIETY NOTES—

Pan-American Medical Congress ... 361

Austin District Medical Society—18th Quarterly Meeting ... 363

S. E. Texas Medical Society Revived ... 364

EDITORIAL—

A Higher Professional Standard ... 365

Ehrlenmeyer's Method ... 367

Homeopathy with a Whiskey-Cure Annex ... 369

Dr. Amick and the Lancet-Clinic ... 370

A Medical Secretary of Public Health ... 371

Ticks and Texas Fever: Dr. Rogers' Paper ... 372

An Unpardonable Liberty ... 373

MEDICAL NEWS AND MISCELLANY ... 374

BOOK NOTICES—

McClelland's Regional Anatomy ... 381

PUBLISHER'S NOTES ... 384

